# Global synthesis indicates widespread occurrence of shifting baseline syndrome

**DOI:** 10.1093/biosci/biae068

**Published:** 2024-08-23

**Authors:** Masashi Soga, Kevin J Gaston

**Affiliations:** Graduate School of Agricultural and Life Sciences at the University of Tokyo, Tokyo, Japan; Environment and Sustainability Institute at the University of Exeter, Penryn, England, United Kingdom

**Keywords:** biodiversity conservation, climate change, environmental degradation, natural resource depletion, policy, pollution

## Abstract

As environmental degradation continues at local, regional, and global levels, people's accepted norms for natural environmental conditions are likely to decline. This phenomenon, known as shifting baseline syndrome (SBS), is increasingly recognized as a likely major obstacle to addressing global environmental challenges. However, the prevalence of SBS remains uncertain. We conducted an extensive systematic review, synthesizing existing research on people's perceived environmental baselines. Our analysis, based on 73 case studies, suggests that SBS is a widespread global phenomenon, occurring across diverse socioeconomic, environmental, and cultural settings. We observed that younger individuals tend to hold lower environmental baselines across various environmental contexts, including climate change, natural resource depletion, biodiversity loss, and pollution. An upward shift in perceived environmental baselines among younger generations was rarely observed. These results underscore the challenge that SBS poses when policy and management responses to environmental degradation are influenced by perceived natural environmental norms.

Our planet is currently experiencing an unprecedented environmental crisis, encompassing a multitude of pressing issues, including climate change, deforestation, biodiversity loss, the depletion of natural resources, and pollution (Díaz et al. 2019, Borrelle et al. 2020, Cheung et al. 2021, Zhou et al. 2023). Mounting evidence suggests that human activities worldwide are pushing the Earth system beyond its planetary boundaries to where the functioning of ecosystems can be dramatically altered, risking the stability of life (Rockström et al. 2009, Steffen et al. 2015, Lade et al. 2020). It is increasingly evident that the speed, magnitude, and ubiquity of these ongoing environmental issues have far-reaching consequences across multiple realms of human activity, posing a significant threat to human health and well-being (Cardinale et al. 2012, Mora et al. 2018, Fuller et al. 2020). However, despite this recognition, the persistence and magnitude of current negative environmental trends continue to be poorly redressed and show few signs of being slowed, let alone halted or reversed (Díaz et al. 2019, Borrelle et al. 2020, Cheung et al. 2021, Zhou et al. 2023). This unsettling reality prompts a fundamental question: Why does society accept and tolerate the ongoing destruction and deterioration of the natural environment, despite the objectively severe circumstances we face?

One possible answer to this question lies in the concept known as *shifting baseline syndrome* (SBS) or *environmental generational amnesia* (Pauly 1995, Kahn 2002, Papworth et al. 2009, Soga and Gaston 2018). SBS refers to the progressive change in accepted norms regarding the state of the natural environment across generations (Pauly 1995, Papworth et al. 2009, Soga and Gaston 2021). Therefore, in a system experiencing impoverishment, people may fail to recognize the extent to which their environment has deteriorated over previous generations (Soga and Gaston 2018, Jones et al. 2020). This lack of awareness can lead to acceptance of incremental environmental degradation, lower expectations for desirable environmental conditions worthy of protection, and decreased support for conservation and management policies (Wu et al. 2011, Plumeridge and Roberts 2017, McClenachan et al. 2018, Jones et al. 2020). Consequently, SBS is increasingly recognized as a potentially significant barrier to promoting and implementing policies and actions aimed at addressing the various global environmental challenges we face today (Soga and Gaston 2018, Mehrabi and Naidoo 2022, Gaston et al. 2023). However, despite its possible importance, understanding of the magnitude and prevalence of this phenomenon remains limited.

To fill this knowledge gap, we conducted a systematic review of research on perceived environmental baselines (the environmental state that individuals use as a reference point for evaluating changes and assessing environmental health). Specifically, we gathered studies that investigated perceived environmental baselines across different age groups and assessed whether younger generations tend to hold lower environmental baselines. Perceived environmental baselines have been studied through two main approaches: directly examining age-related differences in individuals’ perceptions of what environmental conditions are considered normal or healthy and assessing age-related differences in perceived changes in environmental conditions (Soga and Gaston 2018). Our synthesis encompasses studies that explore either approach, acknowledging that the latter is a more indirect one. For the purposes of this study, we define *natural environment conditions* as encompassing all the abiotic and biotic features that constitute ecosystems, including the atmosphere, land, water, flora and fauna. The aims of this review are threefold: characterizing the literature on this topic conducted to date, exploring how geographically widespread are shifts in people's perceived environmental baselines, and identifying key areas for future research that could enhance understanding of SBS.

## Surveying the literature

**Figure 1.**
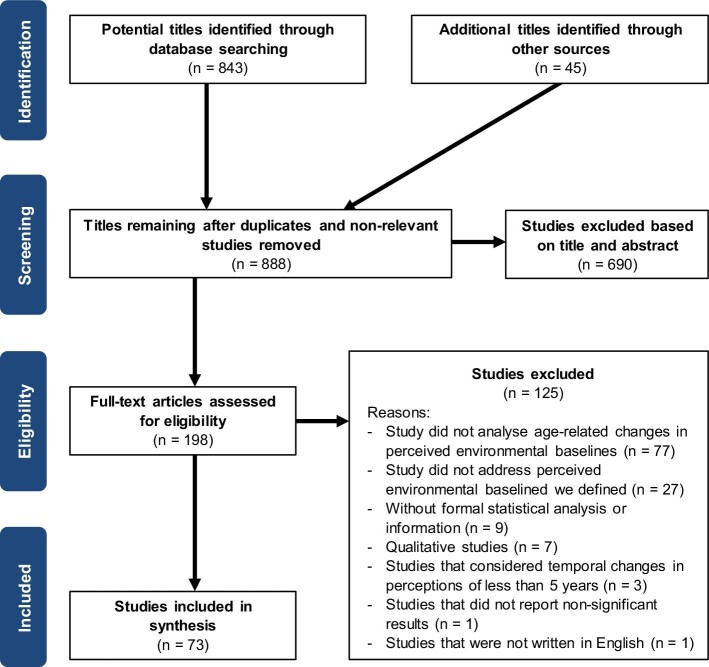
Prisma flow chart (Page et al. 2021) depicting the process and outcome of the literature search.

On 13 March 2023, we conducted a literature search (see figure 1 for the search process and outcomes) using the online database Web of Science. We used the search terms (“perceived change*” OR “perception* of change*”) AND (“environment” OR “climate” OR “biodiversity” OR “ecosystem*” OR “pollution”) OR (“shifting baseline syndrome” OR “environmental generational amnesia” OR “environmental amnesia” OR “shifting baseline*”). The initial search yielded 843 studies (figure 1). During the title and abstract screening stage, we excluded studies that did not focus on perceived environmental baselines, as well as those that were not empirical studies (e.g., meta-analysis, review). Following this screening process, we reviewed the full text of 198 studies (figure 1).

To identify additional studies not captured in the initial literature search, on 30 March 2023, we extended our search to Google Scholar, employing an array of search terms. These terms included 10 combinations related to perceived environmental baselines: *(“perceived changes” AND “environment”, “perception of changes” AND “environment,” “perceived changes” AND “climate,” “perception of changes” AND “climate,” “perceived changes” AND “pollution,” “perception of changes” AND “pollution,” “perceived changes” AND “biodiversity,” “perception of changes” AND “biodiversity,” “perceived changes” AND “ecosystems,” and “perception of changes” AND “ecosystems”)*. They also included four terms associated with SBS: *(“environmental generational amnesia,” “environmental amnesia,” “shifting baseline syndrome,” and “shifting baselines”)*. In addition, we examined the reference lists of reviews specifically focused on perceived environmental baselines (Papworth et al. 2009, Soga and Gaston 2018, Madhuri and Sharma 2020).

### Inclusion and exclusion criteria

Studies were included if they fulfilled the following inclusion and exclusion criteria: They had to investigate the relationship between perceived environmental baselines and people's age, employ a quantitative approach, include a sample of people with an age range of more than 5 years, provide statistical methods or sufficient statistical information regarding age-related differences in perceived environmental baselines, and have a sample size of more than 10 participants. We included studies that used years of experience (e.g., fishing) and explored how different levels of experience relate to variations in perceived environmental conditions. To avoid confounding perceived environmental baselines with proenvironmental attitudes, we excluded studies that were solely focused on measuring people's support and willingness to address environmental issues. One study measured multiple items of environmental perceptions and analyzed them but did not present nonsignificant results. To minimize the potential for publication bias, we excluded this study.

### Data extraction

For each identified study, we recorded the following study characteristics: the author's name, the year of publication, the study location (country or region), the type and number of samples, a measure of age (based on age or years of experience), a measure of perceptions, and the results. We classified the study countries or regions into four categories (low income, low middle income, upper middle income, and high income) based on the World Bank's income classification system (World Bank 2022). The measure of perception was categorized into five distinct groups: changes in climate conditions (encompassing weather patterns such as temperature, rainfall, and wind, as well as environmental changes because of climate change such as drought and flooding), fish abundance (including the abundance and size of fish species primarily used for commercial purposes), animal or plant abundance (encompassing the abundance and size of animal and plant species other than fish used for commercial purposes), land cover (referring to the amount of natural environments such as forests), and pollution (referring to environmental pollution such as air, noise, and water pollution). Although the majority of the identified studies were focused on a single measure of perception, four studies presented results for two perception measures. Subsequently, we treated each measure of perception individually, and the analysis was conducted at the level of each perception measure (see the “Synthesis” section). Furthermore, we assigned each identified study to one of four environmental contexts—biodiversity loss, climate change, environmental degradation, or natural resource depletion—on the basis of the paper's primary focus.

### Synthesis

Given the significant heterogeneity among the included studies in terms of study design, approaches, and outcomes, as well as the limited details provided on statistical analyses, conducting a formal meta-analysis was not feasible. Instead, we synthesized the results using a combination of a vote counting method and a narrative synthesis (Higgins et al. 2019, Campbell et al. 2020). Following the Cochrane Collaboration guidelines for systematic reviews (Higgins et al. 2019), we categorized the results of each perception measure into two groups: older hold higher baselines (indicating a decline in perceived environmental baselines over time) and younger hold higher baselines, without consideration of statistical significance or effect size. For the four studies reporting two perception measures, we documented the results for each measure separately. However, in cases where multiple results were presented within the same perception measure (e.g., perceived changes in different species abundances within the same habitat), we combined them into a single result to avoid issues with pseudoreplication. In the present article, we followed the methodology proposed by Boon and Thomson (2021), categorizing the results as older hold higher baselines if over 70% of the outcomes supported this trend, younger hold higher baselines if over 70% favored this trend, and unclear or conflicting if less than 70% showed a consistent pattern. Duplicate results from repeated analyses (e.g., subgroup analysis) were excluded. In cases where the studies examined both age- and experience-related differences in perceived environmental change, we classified the results as unclear or conflicting if disparities arose between the age and experience analyses. In instances where studies reported both simple correlation and regression model results, our assessment was based on the outcome of the regression model, considering it more reliable because of its ability to control for confounding factors. For studies using regression models, our judgment focused on the main effect of age to streamline result interpretation and disregarded any interaction effects with other variables. The vote-counting results were analyzed using a binomial sign test, assessing the probability of observing the older hold higher baselines direction, with the null hypothesis probability set at .5, in accordance with the Cochrane Collaboration guidelines. The sign test was conducted without considering the studies classified in the unclear or conflicting category.

### Quality assessment

To assess the quality of the identified studies, we applied the Newcastle–Ottawa Quality Assessment Scale, adapted for cross-sectional studies by Modesti and colleagues (2016). This scale evaluates the robustness of a study's results on the basis of three criteria: selection (four items), comparability (one item), and outcome (two items). Given the nature of the research question and our inclusion or exclusion criteria, one of the four items in the selection domain and the items in the outcome domain were deemed not applicable to the selected studies. Therefore, we focused on only the three items in the selection domain and the one item in the comparability domain (see supplement S1). The maximum achievable score is 5, and we classified scores of 4 and 5 as indicative of high quality, 2 and 3 as moderate quality, and 0 and 1 as low quality.

## Overview of the literature

**Figure 2.**
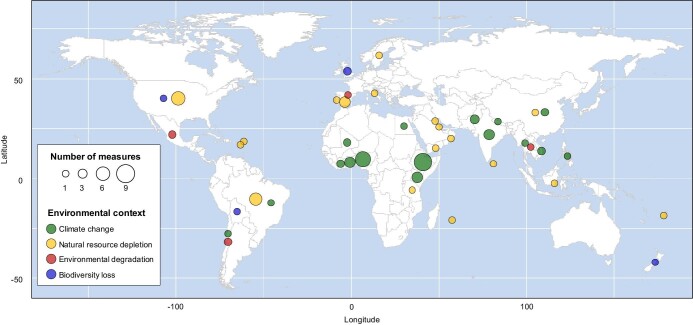
Map displaying the sites of the included studies in our review. The circles on the map indicate the locations of the 77 perception measures from 73 studies (see the “Data extraction” section) included in the analysis, with each circle representing a specific study country or region. The size of the symbols corresponds to the number of perception measures conducted in each country or region. Two studies (Lovell et al. 2020, Almojil 2021) reported results in multiple countries or regions, so multiple symbols are used to show the results separately for each country or region.

Our systematic literature search identified 73 studies containing 77 measures of perception (supplement S2). The perception measures showed substantial variation in terms of the study design, the measure of perceived environmental baselines, the characteristics of the sample, and the study location (supplement S2). They were reported in a wide geographic range, representing 30 countries across six continents (figure 2). The majority of the perception measures (*n* = 42, 54.5%) were focused primarily on climate change as the study context, followed by natural resource depletion (*n* = 423, 19.9%), with a particular emphasis on fisheries (figure 2, table 1). A smaller percentage of perception measures examined environmental degradation (*n* = 4, 9.1%) and biodiversity loss (*n* = 4, 6.5%). Approximately 80% of the perception measures specifically targeted primary industry stakeholders, such as fishers and farmers, whereas the number of studies that investigated other stakeholders, such as policymakers, scientists, and the general population, was much smaller (see supplement S2). Furthermore, around 60% of the perception measures were recorded in middle-income countries, with fewer originating from high- and low-income countries (supplement S2).

**Table 1.** The characteristics of the perception measures (n = 77) categorized into four environmental contexts, based on the geographic region, the income level of the countries or regions where the study was conducted, and the type of samples studied.

		Environmental context							
		Climate change		Natural resource depletion		Environmental degradation		Biodiversity loss	
Study type	Study subcategory	Number of studies	Percentage	Number of studies	Percentage	Number of studies	Percentage	Number of studies	Percentage
Geographic region	Africa	27	64.3	2	8.7	0	0	0	0
	America	2	4.8	9	39.1	4	57.1	2	40.0
	Asia	13	31.0	4	17.4	1	14.3	0	0
	Europe	0	0	7	30.4	2	28.6	2	40.0
	Oceania	0	0	1	4.3	0	0	1	20.0
Income level	Low	10	23.8	0	0	0	0	0	0
	Lower middle	27	64.3	3	13.0	0	0	1	20.0
	Upper middle	4	9.5	9	39.1	1	14.3	0	0
	High	1	2.4	11	47.8	6	85.7	4	80.0
Type of sample	Primary industry stakeholders	39	92.9	21	91.3	0	0	0	0
	Other	3	7.1	2	8.7	7	100	5	100

The overall risk of bias in the included studies was moderate, with an average score of 2.0 (standard deviation = 1.3) out of a maximum of 5.0 (see supplement S3). Concerning selection bias, the majority of studies investigated samples that were at least somewhat representative of the study population (58 out of 73, 75.3%). However, only 15.6% of the studies provided justification for their sample size, and none of them compared responders with nonresponders. Regarding comparability, half of the included studies explored at least one potential confounding factor (e.g., gender) in their analyses.

## Widespread decline in perceived environmental baselines

Among the 77 measures of perceptions identified, 64 measures were categorized into either the older hold higher baselines or the younger hold higher baselines group (see table 2). Out of these, 55 measures were classified as the older hold higher baselines category (85.9%, 95% confidence interval = 75.0–93.4, *p* < .001; table 2). This suggests that, overall, older individuals tended to perceive more environmental changes and have higher environmental baselines than did the younger generations. Indeed, the finding that older individuals hold greater environmental baselines was consistent across various perception outcomes, including changes in fish abundance or size, climate conditions such as temperature and rainfall patterns, the frequency of natural hazards related to climate change (e.g., flooding, drought, increased diseases), wildlife abundance, water resource quality and quantity, and the living conditions of the local environment (supplement S2). This indicates that although SBS has primarily been discussed in the context of fisheries (Pauly 1995, Pinnegar and Engelhard 2008), it is likely associated with diverse environmental issues (Soga and Gaston 2018). Furthermore, the high proportion of studies indicating a decline in perceived environmental baselines was found in regions across a broad range of socioeconomic, environmental, and cultural settings (table 2), highlighting the global nature of SBS and its prevalence across diverse societies.

**Table 2.** The results of the vote counting and the sign test.

						Studies indicating a decline in perceived environmental baselines among younger generations			
Study type	Study subcategory	Number	Older hold higher baselines	Unclear or conflicting	Younger hold higher baselines	Number	Percentage	95% confidence interval	Sign test *p* value
All studies		77	55	13	9	55 of 64	85.9	75.0–93.4	<.001
Environmental contexts	Climate change	42	24	11	7	24 of 31	77.4	58.9–90.4	<.01
	Natural resource depletion	23	22	0	1	22 of 23	95.7	78.1–99.9	<.001
	Environmental degradation	7	6	0	1	6 of 7	85.7	42.1–99.6	.13
	Biodiversity loss	5	3	2	0	–	–	–	–
Geographic region	Africa	29	18	8	3	18 of 21	85.7	63.7–97.0	< 0.01
	America	17	15	1	1	15 of 16	93.8	69.8–99.8	<.001
	Asia	18	12	2	4	12 of 16	75.0	47.6–92.7	.08
	Europe	11	9	1	1	9 of 10	90.0	55.5–99.7	.02
	Oceania	2	1	1	0	–	–	–	–
Income level	Low	10	7	1	2	7 of 9	77.8	40.0–97.2	.18
	Lower middle	31	18	9	4	18 of 22	81.8	59.7–94.8	<.01
	Upper middle	15	14	0	1	14 of 15	93.3	68.1–99.8	< 0.001
	High	20	15	3	2	15 of 17	88.2	63.6–98.5	<.01
Type of sample	Primary industry stakeholders	60	43	10	7	43 of 50	86.0	73.3–94.2	<.001
Other		17	12	3	2	12 of 14	81.8	57.2–98.2	.01

*Note:* The sign test was not performed for the biodiversity loss environmental context or for the Oceania geographic region because of the limited number of perception measures included in these groups.

**Figure 3.**
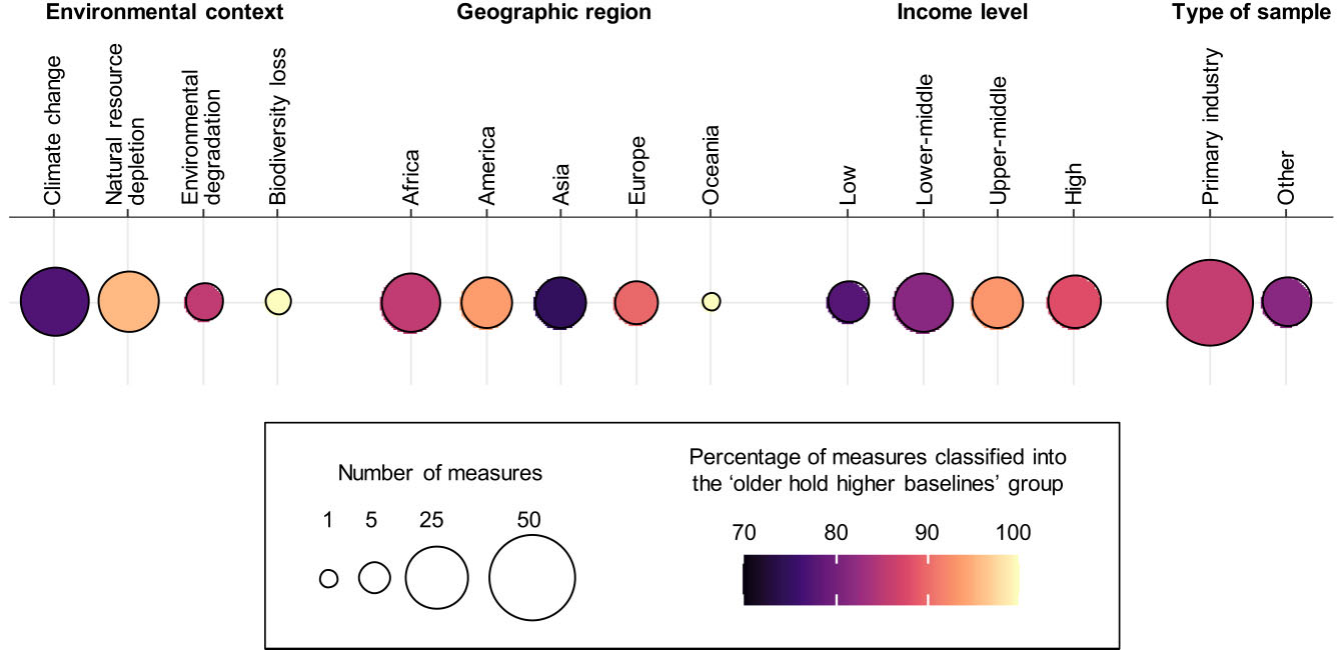
The percentage of perception measures classified into the older hold higher baselines group (see table 2). The results are arranged by environmental contexts, geographical regions, income levels of the country or region, and the type of samples.

The proportion of perception measures classified into the older hold higher baselines category varied across the four environmental contexts addressed (table 2, figure 3). This variation can be attributed to several factors. First, actual levels of environmental degradation likely differed among the contexts. The higher proportion of studies indicating higher environmental baselines among older individuals in the category of natural resource depletion, for example, might be explained by the recent widespread, substantial decline in marine fish abundance (Pacoureau et al. 2021, Yan et al. 2021). Second, the observed differences in perceived environmental baselines may arise from variations in how people perceive environmental degradation in different contexts. The decline in perceived environmental changes can be minimal if the individuals possess a strong understanding of, sensitivity to, and concern for changes in environmental conditions (Shi et al. 2016). Most of the climate change studies were conducted in low-income countries (figure 2, table 1), where heightened sensitivity likely arises because of significant economic implications, such as crop yield reductions (Lee et al. 2015). Third, the variation in reports indicating a decline in perceived environmental baselines among younger generations across the four environmental contexts might be caused partly by publication bias (Franco et al. 2014). Research in the fisheries field, where SBS was initially proposed (Pauly 1995), often emphasized exploring age- or experience-related differences in perceived environmental baselines as a primary research objective. Consequently, studies in this field that failed to identify a clear age or experience effect might not have been published.

In our systematic review, several case studies quantified the magnitude of the decline in perceived environmental baselines among the younger generations, although there was a lack of consistency in the methodological approaches employed. The findings suggest significant variability in the magnitude of these declines across different cases. For example, Alessa and colleagues (2008) reported that the proportion of individuals in Alaska perceiving deterioration in water quality and decreased water availability decreased by over 70% in the younger generations compared with the older generations. Similarly, in Italy, Veneroni and Fernandes (2021) found that the number of benthic species perceived as depleted by fishers was more than 80% smaller in the youngest generation compared with the oldest one. Conversely, studies that observed a less severe pattern (e.g., Manandhar et al. 2015, Funatsu et al. 2019) or almost no change (e.g., Shitu et al. 2018, Tofu 2018) also exist. We were unable to formally quantify the overall magnitude and variability of these declines because of limited data availability. Nevertheless, exploring the magnitude and rate of SBS, along with the factors influencing variations in pace, remains a crucial research challenge.

Although the observed greater environmental baselines among older generations in this study suggest the occurrence of SBS, we acknowledge the potential for biases. One such bias is the memory illusion effect, which proposes that older individuals may inaccurately recall past environmental conditions, perceiving change where this was very limited or absent (see Papworth et al. 2009). The extent of this effect can be assessed with precise information on the actual historical environmental conditions in each study site analyzed by the selected articles. Unfortunately, however, the majority of the studies did not provide such detailed information, although some did (e.g., Roco et al. 2015, Shrestha et al. 2019, Jones et al. 2020, Teshome et al. 2021). Consequently, we were unable to determine how this effect influenced our results. Nevertheless, considering that almost all of the included studies were conducted in areas where environmental degradation is actually occurring, it is unlikely that such an effect had a strong impact.

In our analysis of 77 perception measures, only a limited subset (9 out of 77 case studies; table 2) falls under the younger hold higher baselines category, and the majority of these did not yield statistically significant results. Although this observation suggests that upward shifts in people's perceived environmental baselines may be much less common than downward shifts, it does not rule out the possibility of the former happening. Indeed, although SBS was initially conceived to explain the downward shift of people's perceptions and accepted norms toward natural environmental conditions (Pauly 1995, Papworth et al. 2009, Soga and Gaston 2018), theoretically, these baselines can also shift in an upward direction (Steen and Jachowski 2013). Because of the methodological limitations associated with the vote counting approach, we were not able to determine whether the low proportion of case studies in the younger hold higher baselines category is because this trend is indeed rare or because researchers have not explored this possibility or have not published results showing this trend in peer-reviewed journals (i.e., are indicative of publication bias). Although the current discourse on SBS is primarily focused on environmental degradation, future research should also explore this phenomenon in the context of environmental improvement.

## Implications

Perceptions and awareness of environmental issues are known to be key predictors of people's willingness to support and engage in practices and policies aimed at addressing these issues (Halady and Rao 2010, Spence et al. 2011, Masud et al. 2015, Arı and Yılmaz 2017). The widespread decline in perceived environmental baselines found in the present study is therefore of profound concern (Pinnegar and Engelhard 2008, Soga and Gaston 2018, Gaston et al. 2023). It may lead policymakers and resource managers to establish and use inappropriate (i.e., less ambitious) targets for environmental conservation and restoration programs (Humphries and Winemiller 2009, Bonebrake et al. 2010, Plumeridge and Roberts 2017, Joy and Canning 2020, Mehrabi and Naidoo 2022, Scopes et al. 2023, but see Jones et al. 2021) and to undermine broader public support for more ambitious targets when these are proposed (Wu et al. 2011, Roman et al. 2015, McClenachan et al. 2018, Jones et al. 2020, Lovell et al. 2020). With ongoing increases in global human population and per capita demand for natural resources, the pace of environmental degradation is accelerating rapidly worldwide (Díaz et al. 2019, Borrelle et al. 2020, Cheung et al. 2021, Zhou et al. 2023), potentially making SBS a greater issue and its impacts even more marked.

Given the profound implications of SBS for environmental conservation and management, it seems crucial to consider how to address this issue effectively. Arguably, the most direct strategy to achieve this objective is the restoration of natural environments, even if only locally achievable (Soga and Gaston 2018). With suitable opportunities, this approach can provide people with personal, direct experiences and vivid memories of natural environments that exhibit relatively high quality (Akhshik et al. 2023). Encouragingly, many parts of the world have experienced some local environmental improvement recently. Certain European and North American countries, for example, have implemented rewilding practices as major conservation strategies, resulting in the recovery of rare and locally extinct wildlife species (Lorimer et al. 2015, Svenning et al. 2016). In addition, many higher-income and urbanized societies have dedicated substantial efforts to enhancing environmental conditions, including advances in air and water quality and the expansion of greenspace coverage (e.g., Tan et al. 2013, Ma et al. 2020, Wu et al. 2021).

This said, given the existing prevalence and escalating scale of environmental degradation caused by human activities, relying solely on people's experiences of environmental restoration will not be sufficient to counter SBS (Soga and Gaston 2018). Indeed, many ecosystems on Earth have long been experiencing significant human-driven anthropogenic impacts, making it challenging and, in some cases, impossible to restore them to their original state, especially as they have transitioned into what has been referred to as Anthropocene baselines (Kopf et al. 2015). In addition, there are some environmental challenges, such as climate change and the depletion of fish abundance, where even with immediate implementation of environmental restoration activities, marked immediate responses may not be feasible. In such cases, the provision of accurate information about past environmental conditions becomes virtually the only viable option to enhance people's perceived environmental baselines (Baisre 2013, Plumeridge and Roberts 2017). Indeed, there is a growing effort to reconstruct precise historical conditions of the natural environment—specifically, those predating large-scale anthropogenic activities—using available ecological, historical, and archaeological data, coupled with robust statistical approaches (e.g., Lotze and Worm 2009, Bonebrake et al. 2010, Baisre 2013, Rodrigues et al. 2019, Collins et al. 2020).

The delivery of environmental information is feasible across various settings, including educational and recreational activities conducted in schools or museums (Lindemann-Matthies 2002, Moss et al. 2017, Rees 2017). These activities can also contribute to decreasing people's psychological distance to environmental issues and can enhance general environmental literacy (Lee et al. 2015, Weber 2016). However, it is important to acknowledge that, although information provision is generally more immediately achievable than environmental restoration, its effectiveness often proves limited and short lived (Otto and Pensini 2017). Therefore, future research should be focused on maximizing the environmental outcomes of information provision. Recent technological advances in virtual reality, for example, provide a more immersive experience closely resembling the real world, which has the potential to enhance the effectiveness of information provision (Markowitz et al. 2018, Nelson et al. 2020, Meijers et al. 2023).

In addition to providing direct experiences and information regarding natural environments, as was discussed above, employing more social approaches would also be crucial in helping people understand the scale of ongoing environmental issues and their societal impacts. Indeed, people's perceptions of environmental issues are known to be shaped by a wide range of personal, social, cultural, and political factors (see Lee et al. 2015, Weber 2016 for the case of climate change). Therefore, future policies and actions aimed at reversing SBS should combine multiple strategies rather than solely relying on providing experiences and knowledge, although these remain significant.

## Future research directions

Following our literature search and systematic review, we have identified several crucial areas for future research. First, there is a need more commonly to broaden the study of SBS beyond the realms of climate change and fisheries. The studies to date have predominantly been focused on these domains (table 1), leaving the prevalence of SBS in other environmental contexts somewhat unclear. However, given the alarming rates of diverse environmental challenges beyond climate change and fish stock decline, it is reasonable to assume that people's perceived environmental baselines could be declining across a much broader spectrum of environmental contexts than those we focused on in our review.

Second, although we observed declines in perceived environmental baselines across various countries, the research efforts showed a geographical bias. For example, climate change research was mainly concentrated in Africa and Asia (figure 2). Given that people's perceptions of environmental change can be shaped by socioeconomic and cultural factors, future studies should investigate SBS patterns in a more diverse range of global populations.

Third, our literature review highlighted a bias toward research involving primary industry stakeholders, such as farmers and fishers (table 1). Although this focus is understandable because of the significant environmental impact and economic dependence of these stakeholders, it is crucial to expand this scope. Other groups, including laypeople, policymakers, and practitioners, also play substantial roles in shaping environmental outcomes through their decisions and actions (Beckage et al. 2018, Nielsen et al. 2021). Therefore, future research on SBS should encompass these diverse stakeholders to achieve a comprehensive understanding of its dynamics and implications.

Fourth, our literature review underscored the importance of longitudinal studies that track people's perceived environmental baselines over time. Our review exclusively encompassed cross-sectional studies that investigated perceived environmental baselines across different age groups at a single point in time. The absence of longitudinal studies is noteworthy because it hinders the possibility of ascertaining the extents to which lower environmental baselines among younger age groups identified in this study are influenced by a longitudinal effect, where perceived environmental baselines in a population indeed decline over time or by a cohort effect, where such baselines in a population remain stable over time and variations across ages stem from generational differences in personal, social, and environmental conditions.

Fifth, despite our presenting the first systematic review on this topic, it is crucial that a body of studies is generated that enables more quantitative analysis, such as a meta-analysis, in the future. Because of the heterogeneity of the included studies, we employed a vote-counting approach. Although this method is commonly used in such situations (Higgins et al. 2019, Campbell et al. 2020), it has several limitations. For instance, it does not account for the quality of studies and sample sizes, which varied substantially among the included studies (see supplement S2). It also does not allow for consideration of the variation in effect sizes—that is, the magnitude of changes in people's perceived environmental baselines. More quantitative studies will enable synthesis approaches that provide crucial insights into the drivers of SBS and how to address it.

## Conclusions

Environmental scientists have extensively documented the rapid and ongoing decline of natural environmental conditions. Our review, based on the available evidence, confirms that this downward trend is evident also within people's minds. Indeed, it suggests that people's perceived environmental baselines are experiencing widespread decline. With the findings of our study in mind, the next key step in this area is to assess how and to what extent this widespread decline in perceived environmental baselines affects efforts to address ongoing environmental issues. Given the potentially diverse ways SBS can influence nature conservation and management (from changes in individual daily behavior to policymaking), its impact cannot be overlooked and may be profound.

## Supplementary Material

biae068_Supplemental_Files

## Author Biography

Masashi Soga (asoga@g.ecc.u-tokyo.ac.jp) is affiliated with the Graduate School of Agricultural and Life Sciences at the University of Tokyo, in Tokyo, Japan. Kevin J. Gaston is affiliated with the Environment and Sustainability Institute at the University of Exeter, in Penryn, England, in the United Kingdom.
