# Supplementary material for: Global synthesis indicates widespread occurrence of shifting baseline syndrome
Source: Bioscience. 2024 Aug 23;74(10):686–94. doi: 10.1093/biosci/biae068 (PMC11494512; doi:10.1093/biosci/biae068)
Supplement: biae068_Supplemental_Files [file biae068_supplemental_files.zip › Appendix 1.docx]

**Supplementary Appendix S1. NEWCASTLE-OTTAWA QUALITY ASSESSMENT SCALE (adapted for cross-sectional studies)**

**Selection:** (Maximum 5 stars)

1) Representativeness of the sample:

a) Truly representative of the average in the target population. * (all subjects or random sampling)

b) Somewhat representative of the average in the target population. * (nonrandom sampling)

c) Selected group of users.

d) No description of the sampling strategy.

2) Sample size:

a) Justified and satisfactory. *

b) Not justified.

3) Non-respondents:

a) Comparability between respondents and non-respondents characteristics is established, and the response rate is satisfactory. *

b) The response rate is unsatisfactory, or the comparability between respondents and non-respondents is unsatisfactory.

c) No description of the response rate or the characteristics of the responders and the non-responders.

**Comparability:** (Maximum 2 stars)

1) The subjects in different outcome groups are comparable, based on the study design or analysis. Confounding factors are controlled.

a) The study controls for the most important factor (select one). *

b) The study control for any additional factor. *
