# Supplementary material for: Global synthesis indicates widespread occurrence of shifting baseline syndrome
Source: Bioscience. 2024 Aug 23;74(10):686–94. doi: 10.1093/biosci/biae068 (PMC11494512; doi:10.1093/biosci/biae068)
Supplement: biae068_Supplemental_Files [file biae068_supplemental_files.zip › Appendix 2.docx]

**Supplementary Appendix S2. List of 73 case studies (77 measures) included in the review.**

| **Study** | **Year published** | **Country** | **Region** | **Income level** | **Type of sample** | **Sample size** | **Measure of time** | **Measure of perceived environmental condition** | **Number of measures included** | **Context** | **Results** |
| --- | --- | --- | --- | --- | --- | --- | --- | --- | --- | --- | --- |
| Agwu et al. (2018) | 2018 | Nigeria | Africa | Low middle | Farmers | 215 | Age | Climate conditions | 5 | Climate change | Unclear/conflicting |
| Ainsworth et al. (2008) | 2008 | Indonesia | Asia | Low middle | Fishers | 209 | Years of fishing | Fish abundance | 6 | Natural resource depletion | Older hold higher baselines |
| Akano et al. (2023) | 2022 | Nigeria | Africa | Low middle | Farmers | 400 | Age; Years of farming | Climate conditions | 1 | Climate change | Unclear/conflicting |
| Alessa et al. (2008) | 2008 | US | America | High | Local residents | 103 | Age | Pollution | 2 | Natural resource depletion | Older hold higher baselines |
| Alfonso et al. (2017) | 2017 | Chile | America | High | Local residents | 78 | Age | Land cover | 1 | Environmental degradation | Older hold higher baselines |
| Alfonso et al. (2017) | 2017 | Chile | America | High | Local residents | 78 | Age | Pollution | 2 | Environmental degradation | Younger hold higher baselines |
| Ali et al. (2017) | 2017 | Pakistan | Asia | Low middle | Farmers | 500 | Age | Climate conditions | 3 | Climate change | Older hold higher baselines |
| Almojil (2021) | 2021 | State of Kuwait; Kingdom of Bahrain; Sultanate of Oman; Republic of Yemen | Asia | NA | Fishers | 22 | Years of fishing | Fish abundance | 1 | Natural resource depletion | Older hold higher baselines |
| Amadou et al. (2015) | 2015 | Ghana | Africa | Low middle | Farmers | 186 | Age; Years of farming | Climate conditions | 1 | Climate change | Unclear/conflicting |
| Amoutchi et al. (2021) | 2021 | Cote d’Ivoire | Africa | Low middle | Fishers | 381 | Age | Climate conditions | 4 | Climate change | Older hold higher baselines |
| Amoutchi et al. (2021) | 2021 | Cote d’Ivoire | Africa | Low middle | Fishers | 381 | Age | Fish abundance | 1 | Climate change | Older hold higher baselines |
| Ansari et al. (2018) | 2018 | India | Asia | Low middle | Farmers | 120 | Age; Years of farming | Climate conditions | 1 | Climate change | Younger hold higher baselines |
| Apata (2011) | 2011 | Nigeria | Africa | Low middle | Farmers | 350 | Age; Years of farming | Climate conditions | 1 | Climate change | Older hold higher baselines |
| Assaye (2016) | 2016 | Ethiopia | Africa | Low | Farmers | 156 | Age | Climate conditions | 1 | Climate change | Older hold higher baselines |
| Ayal and Leal Filho (2017) | 2017 | Ethiopia | Africa | Low | Farmers | 250 | Age | Climate conditions | 3 | Climate change | Older hold higher baselines |
| Bao and Drew (2016) | 2016 | Fiji | Oceania | Upper middle | Fishers | 33 | Age | Fish abundance | 1 | Natural resource depletion | Older hold higher baselines |
| Barbosa-Filho et al. (2020) | 2020 | Brazil | America | Upper middle | Fishers | 222 | Age | Fish abundance | 1 | Natural resource depletion | Older hold higher baselines |
| Bender et al. (2013) | 2013 | Brazil | America | Upper middle | Fishers | 53 | Age | Fish abundance | 1 | Natural resource depletion | Older hold higher baselines |
| Bender et al. (2014) | 2014 | Brazil | America | Upper middle | Fishers | 214 | Years of fishing | Fish abundance | 6 | Natural resource depletion | Older hold higher baselines |
| Bobadoye et al. (2020) | 2020 | Nigeria | Africa | Low middle | Farmers | 120 | Age; Years of farming | Climate conditions | 1 | Climate change | Older hold higher baselines |
| Braga et al. (2022) | 2022 | Portugal | Europe | High | Fishers | 50 | Years of fishing | Fish abundance | 1 | Natural resource depletion | Older hold higher baselines |
| Bunce et al. (2008) | 2008 | Mauritius | Africa | Upper middle | Fishers | 93 | Age | Fish abundance | 1 | Natural resource depletion | Older hold higher baselines |
| Coster and Adeoti (2021) | 2021 | Nigeria | Africa | Low middle | Farmers | 346 | Age; Years of farming | Climate conditions | 1 | Climate change | Older hold higher baselines |
| Debela et al. (2015) | 2015 | Ethiopia | Africa | Low | Farmers | 475 | Age | Climate conditions | 1 | Climate change | Older hold higher baselines |
| Deressa et al. (2011) | 2011 | Ethiopia | Africa | Low | Farmers | 608 | Age | Climate conditions | 1 | Climate change | Older hold higher baselines |
| Fatuase et al. (2014) | 2014 | Nigeria | Africa | Low middle | Farmers | 135 | Age; Years of farming | Climate conditions | 1 | Climate change | Unclear/conflicting |
| Fernández-Llamazares et al. (2015) | 2015 | Bolivia | America | Low middle | Indigenous people | 300 | Age | Animal/plant abundance | 4 | Biodiversity loss | Older hold higher baselines |
| Frezza and Clem (2015) | 2015 | US | America | High | Fishers | 64 | Years of fishing | Fish abundance | 1 | Natural resource depletion | Older hold higher baselines |
| Funatsu et al. (2019) | 2019 | Brazil | America | Upper middle | Local residents | 1244 | Age | Climate conditions | 4 | Climate change | Older hold higher baselines |
| Giglio et al. (2015) | 2015 | Brazil | America | Upper middle | Fishers | 102 | Age | Fish abundance | 2 | Natural resource depletion | Older hold higher baselines |
| Guodaar et al. (2017) | 2017 | Ghana | Africa | Low middle | Farmers | 378 | Age | Climate conditions | 3 | Climate change | Unclear/conflicting |
| Habtemariam et al. (2016) | 2016 | Ethiopia | Africa | Low | Farmers | 182 | Age | Climate conditions | 2 | Climate change | Older hold higher baselines |
| Jabik et al. (2022) | 2022 | Ghana | Africa | Low middle | Farmers | 350 | Age | Climate conditions | 1 | Climate change | Older hold higher baselines |
| Jones et al. (2020) | 2020 | UK | Europe | High | Local residents | 282 | Age | Animal/plant abundance | 2 | Biodiversity loss | Unclear/conflicting |
| Katikiro (2014) | 2014 | Tanzania | Africa | Low middle | Fishers | 350 | Age | Fish abundance | 2 | Natural resource depletion | Older hold higher baselines |
| Kawadia and Tiwari (2017) | 2017 | India | Asia | Low middle | Farmers | 470 | Age | Climate conditions | 4 | Climate change | Older hold higher baselines |
| Kidanu et al. (2016) | 2016 | Ethiopia | Africa | Low | Farmers | 171 | Age | Climate conditions | 1 | Climate change | Younger hold higher baselines |
| Lasco et al. (2016) | 2016 | Philippines | Asia | Low middle | Farmers | 636 | Age | Climate conditions | 2 | Climate change | Unclear/conflicting |
| Leitao et al. (2020) | 2020 | Spain | Europe | High | Fishers | 49 | Age | Fish abundance | 1 | Natural resource depletion | Older hold higher baselines |
| Li et al. (2013) | 2013 | China | Asia | Upper middle | Farmers | 100 | Age | Climate conditions | 1 | Climate change | Older hold higher baselines |
| Lovell et al. (2020) | 2020 | UK Overseas Territory (Montserrat)/ Antigua and Barbuda | Europe | High | Fishers | 40 | Years of fishing | Fish abundance | 1 | Natural resource depletion | Older hold higher baselines |
| Lozano-Montes et al. (2008) | 2008 | US | America | High | Fishers | 49 | Age | Fish abundance | 1 | Natural resource depletion | Older hold higher baselines |
| Lyver et al. (2021) | 2021 | New Zealand | Oceania | High | Indigenous people | 43 | Age | Animal/plant abundance | 6 | Biodiversity loss | Unclear/conflicting |
| Magadán-Díaz and Rivas-García (2022) | 2022 | Spain | Europe | High | Local residents | 382 | Age; Year of residing | Pollution | 1 | Environmental degradation | Older hold higher baselines |
| Manandhar et al. (2015) | 2015 | Thailand | Asia | Upper middle | Local residents | 87 | Age | Climate conditions | 1 | Climate change | Older hold higher baselines |
| Mata-Lara et al. (2018) | 2018 | Mexico | America | Upper middle | Local residents | 150 | Years of staying | Fish abundance | 2 | Environmental degradation | Older hold higher baselines |
| Mata-Lara et al. (2018) | 2018 | Mexico | America | Upper middle | Local residents | 150 | Years of staying | Pollution | 1 | Environmental degradation | Older hold higher baselines |
| McClenachan and Neal (2023) | 2023 | US | America | High | Fishers | 23 | Years of fishing | Fish abundance | 2 | Natural resource depletion | Older hold higher baselines |
| Mishra and Pede (2017) | 2017 | Vietnam | Asia | Low middle | Farmers | 214 | Age of household head; Years of farming | Climate conditions | 1 | Climate change | Older hold higher baselines |
| Muldrow et al. (2020) | 2020 | US | America | High | Scientists | 54 | Age; Years of dive experience | Animal/plant abundance | 1 | Biodiversity loss | Older hold higher baselines |
| Mustafa et al. (2018) | 2018 | Pakistan | Asia | Low middle | Farmers | 224 | Years of farming | Climate conditions | 1 | Climate change | Older hold higher baselines |
| Mwalusepo et al. (2015) | 2015 | Kenya | Africa | Low middle | Farmers | 510 | Age | Climate conditions | 1 | Climate change | Older hold higher baselines |
| Ndambiri et al. (2013) | 2013 | Kenya | Africa | Low middle | Farmers | 246 | Age; Years of farming | Climate conditions | 1 | Climate change | Older hold higher baselines |
| Nyang'au et al. (2021) | 2021 | Kenya | Africa | Low middle | Farmers | 196 | Age | Climate conditions | 3 | Climate change | Unclear/conflicting |
| Ochieng et al. (2017) | 2017 | Kenya | Africa | Low middle | Farmers | 1309 | Age | Climate conditions | 2 | Climate change | Younger hold higher baselines |
| Papworth et al. (2009) | 2009 | UK | Europe | High | Local residents | 50 | Age | Animal/plant abundance | 4 | Biodiversity loss | Older hold higher baselines |
| Pita et al. (2020) | 2020 | Spain | Europe | High | Fishers | 14 | Years of fishing | Fish abundance | 1 | Natural resource depletion | Older hold higher baselines |
| Pouso et al. (2018) | 2018 | Spain | Europe | High | Recreational fishers | 146 | Years of fishing | Pollution | 1 | Environmental degradation | Older hold higher baselines |
| Pouso et al. (2018) | 2018 | Spain | Europe | High | Recreational fishers | 146 | Years of fishing | Fish abundance | 3 | Natural resource depletion | Younger hold higher baselines |
| Raghuvanshi et al. (2017) | 2017 | India | Asia | Low middle | Farmers | 110 | Age | Climate conditions | 1 | Climate change | Younger hold higher baselines |
| Raza et al. (2022) | 2022 | Pakistan | Asia | Low middle | Farmers | 140 | Age | Climate conditions | 3 | Climate change | Younger hold higher baselines |
| Roco et al. (2015) | 2015 | Chile | America | High | Farmers | 274 | Age; Years of farming | Climate conditions | 1 | Climate change | Unclear/conflicting |
| Sanogo et al. (2017) | 2017 | Mali | Africa | Low | Farmers | 240 | Age; Years of farming | Climate conditions | 12 | Climate change | Younger hold higher baselines |
| Shitu et al. (2018) | 2018 | Nigeria | Africa | Low middle | Farmers | 180 | Age; Years of farming | Climate conditions | 1 | Climate change | Unclear/conflicting |
| Shrestha et al. (2019) | 2019 | Nepal | Asia | Low middle | Local residents | 5060 | Age; Years of residing | Climate conditions | 1 | Climate change | Unclear/conflicting |
| Song et al. (2019) | 2019 | China | Asia | Upper middle | Farmers | 1350 | Years of farming | Climate conditions | 2 | Climate change | Younger hold higher baselines |
| Tesfahunegn et al. (2016) | 2016 | Ethiopia | Africa | Low | Farmers | 60 | Age | Climate conditions | 1 | Climate change | Older hold higher baselines |
| Teshome et al. (2021) | 2021 | Ethiopia | Africa | Low | Farmers | 364 | Age | Climate conditions | 2 | Climate change | Older hold higher baselines |
| Teye and Yaro (2015) | 2014 | Ghana | Africa | Low middle | Farmers | 530 | Age | Climate conditions | 1 | Climate change | Older hold higher baselines |
| Thi Lan Huong et al. (2017) | 2017 | Vietnam | Asia | Low middle | Farmers | 335 | Years of farming | Climate conditions | 5 | Climate change | Older hold higher baselines |
| Tofu (2018) | 2018 | Ethiopia | Africa | Low | Farmers | 241 | Age; Years of farming | Climate conditions | 2 | Climate change | Unclear/conflicting |
| Tuntipisitkul et al. (2021) | 2021 | Thailand | Asia | Upper middle | Local residents | 495 | Age | Pollution | 1 | Environmental degradation | Older hold higher baselines |
| Turvey et al. (2010) | 2010 | China | Asia | Upper middle | Fishers | 599 | Age | Fish abundance | 2 | Natural resource depletion | Older hold higher baselines |
| Veneroni and Fernandes (2021) | 2021 | Italy | Europe | High | Fishers | 53 | Age | Fish abundance | 2 | Natural resource depletion | Older hold higher baselines |
| Venkatachalam et al. (2010) | 2010 | Sri Lanka | Asia | Low middle | Fishers | 120 | Age | Fish abundance | 1 | Natural resource depletion | Older hold higher baselines |
| Zapelini et al. (2020) | 2020 | Brazil | America | Upper middle | Fishers | 188 | Years of fishing | Fish abundance | 1 | Natural resource depletion | Older hold higher baselines |
| van den Heuvel and Rönnbäck (2023) | 2023 | Sweden | Europe | High | Fishers | 277 | Years of fishing | Fish abundance | 2 | Natural resource depletion | Older hold higher baselines |

**References**

Agwu, O. P., Bakayoko, A., Jimoh, S. O., & Stefan, P. (2018). Farmers’ perceptions on cultivation and the impacts of climate change on goods and services provided by Garcinia kola in Nigeria. Ecological Processes, 7, 1-10.

Ainsworth, C. H., Pitcher, T. J., & Rotinsulu, C. (2008). Evidence of fishery depletions and shifting cognitive baselines in Eastern Indonesia. Biological Conservation, 141, 848-859.

Akano, O., Modirwa, S., Oluwasemire, K., & Oladele, O. (2023). Awareness and perception of climate change by smallholder farmers in two agroecological zones of Oyo state Southwest Nigeria. *GeoJournal*, 88, 39-68.

Ali, A. (2017). Coping with climate change and its impact on productivity, income, and poverty: evidence from the Himalayan region of Pakistan. International journal of disaster risk reduction, 24, 515-525.

Alessaa, L., Kliskey, A., Williams, P., & Barton, M. (2008). Perception of change in freshwater in remote resource-dependent Arctic communities. Global Environmental Change, 18, 153-164.

Alfonso, A., Zorondo-Rodríguez, F., & Simonetti, J. A. (2017). Perceived changes in environmental degradation and loss of ecosystem services, and their implications in human well-being. International Journal of Sustainable Development & World Ecology, 24, 561-574.

Almojil, D. (2021). Local ecological knowledge of fisheries charts decline of sharks in data-poor regions. Marine Policy, 132, 104638.

Amadou, M. L., Villamor, G. B., Attua, E. M., & Traoré, S. B. (2015). Comparing farmers’ perception of climate change and variability with historical climate data in the Upper East Region of Ghana. Ghana Journal of Geography, 7, 47-74.

Amoutchi, A. I., Mehner, T., Ugbor, O. N., Kargbo, A., & Paul, K. E. (2021). Fishermen’s perceptions and experiences toward the impact of climate change and anthropogenic activities on freshwater fish biodiversity in Côte d’Ivoire. Discover Sustainability, 2, 56.

Ansari, M. A., Joshi, S., & Raghuvanshi, R. (2018). Understanding farmers perceptions about climate change: a study in a North Indian State. Advances in Agriculture and Environmental Science, 1, 85-89.

Apata, T. G. (2011). Factors influencing the perception and choice of adaptation measures to climate change among farmers in Nigeria. Evidence from farming households in Southwest Nigeria. Environmental Economics, 2, 74-83.

Assaye, A. (2016). Smallholder farmers’ perceptions to climate change: the case of Ankesha Guagusa District of Awi Zone, north western Ethiopia. Research Journal of Agricultural and Environment Science, 3, 1-14.

Ayal, D. Y., & Leal Filho, W. (2017). Farmers’ perceptions of climate variability and its adverse impacts on crop and livestock production in Ethiopia. Journal of Arid Environments, 140, 20-28.

Bao, K., & Drew, J. (2016). Traditional ecological knowledge, shifting baselines, and conservation of Fijian molluscs. Pacific Conservation Biology, 23, 81-87.

Barbosa-Filho, M. L., Seminara, C. I., Tavares, D. C., Siciliano, S., Hauser-Davis, R. A., & da Silva Mourão, J. (2020). Artisanal fisher perceptions on ghost nets in a tropical South Atlantic marine biodiversity hotspot: Challenges to traditional fishing culture and implications for conservation strategies. Ocean & Coastal Management, 192, 105189.

Bender, M. G., Floeter, S. R., & Hanazaki, N. (2013). Do traditional fishers recognise reef fish species declines? Shifting environmental baselines in E astern Brazil. Fisheries Management and Ecology, 20, 58-67.

Bender, M. G., Machado, G. R., Silva, P. J. D. A., Floeter, S. R., Monteiro-Netto, C., Luiz, O. J., & Ferreira, C. E. (2014). Local ecological knowledge and scientific data reveal overexploitation by multigear artisanal fisheries in the Southwestern Atlantic. PLoS One, 9, e110332.

Bobadoye, B., Jimoh, K., Bobadoye, A., & Aluko, O. J. (2020). Agroforestry farmers’ perception of climate change in Ibadan, Nigeria. Journal of Research in Forestry, Wildlife and Environment, 12, 22-30.

Braga, H. O., Bender, M. G., Oliveira, H. M., Pereira, M. J., & Azeiteiro, U. M. (2022). Fishers’ knowledge on historical changes and conservation of Allis shad-*Alosa alosa* (Linnaeus, 1758) in Minho River, Iberian Peninsula. Regional Studies in Marine Science, 49, 102094.

Bunce, M., Rosendo, S., & Brown, K. (2010). Perceptions of climate change, multiple stressors and livelihoods on marginal African coasts. Environment, Development and Sustainability, 12, 407-440.

Coster, A. S., & Adeoti, A. I. (2020). Analysis of perception and adaptation of maize–based farming households to climate change in Nigeria. Tropical Agriculture, 97, 268-280.

Debela, N., Mohammed, C., Bridle, K., Corkrey, R., & McNeil, D. (2015). Perception of climate change and its impact by smallholders in pastoral/agropastoral systems of Borana, South Ethiopia. SpringerPlus, 4, 1-12.

Deressa, T. T., Hassan, R. M., & Ringler, C. (2011). Perception of and adaptation to climate change by farmers in the Nile basin of Ethiopia. The Journal of Agricultural Science, 149, 23-31.

Fatuase, A., & Ajibefun, I. (2014). Perception and adaptation to climate change among farmers in selected communities of Ekiti State, Nigeria. Journal of Agricultural Faculty of Gaziosmanpaşa University, 31, 100-113.

Fernández-Llamazares, Á., Méndez-López, M. E., Díaz-Reviriego, I., McBride, M. F., Pyhälä, A., Rosell-Melé, A., & Reyes-García, V. (2015). Links between media communication and local perceptions of climate change in an indigenous society. Climatic Change, 131, 307-320.

Frezza, P. E., & Clem, S. E. (2015). Using local fishers’ knowledge to characterize historical trends in the Florida Bay bonefish population and fishery. Environmental Biology of Fishes, 98, 2187-2202.

Funatsu, B. M., Dubreuil, V., Racapé, A., Debortoli, N. S., Nasuti, S., & Le Tourneau, F. M. (2019). Perceptions of climate and climate change by Amazonian communities. Global Environmental Change, 57, 101923.

Giglio, V. J., Luiz, O. J., & Schiavetti, A. (2015). Marine life preferences and perceptions among recreational divers in Brazilian coral reefs. Tourism Management, 51, 49-57.

Guodaar, L., Beni, A., & Benebere, P. (2017). Using a mixed-method approach to explore the spatiality of adaptation practices of tomato farmers to climate variability in the Offinso North District, Ghana. Cogent Social Sciences, 3, 1273747.

Habtemariam, L. T., Gandorfer, M., Kassa, G. A., & Heissenhuber, A. (2016). Factors influencing smallholder farmers’ climate change perceptions: a study from farmers in Ethiopia. Environmental Management, 58, 343-358.

Jabik, B. B., Bawakyillenuo, S., & Codjoe, S. N. (2022). Perceived effects of climate change on local knowledge of small-scale farmers in the Garu-Tempane District of Ghana. The International Journal of Climate Change: Impacts and Responses, 15, 85.

Jones, L. P., Turvey, S. T., Massimino, D., & Papworth, S. K. (2020). Investigating the implications of shifting baseline syndrome on conservation. People and Nature, 2, 1131-1144.

Katikiro, R. E. (2014). Perceptions on the shifting baseline among coastal fishers of Tanga, Northeast Tanzania. Ocean & Coastal Management, 91, 23-31.

Kawadia, G., & Tiwari, E. (2017). Farmers’ perception of climate change in Madhya Pradesh. Area Development and Policy, 2, 192-207.

Kidanu, A., Kibret, K., Hajji, J., Mohammed, M., & Ameha, Y. (2016). Farmers perception towards climate change and their adaptation measures in Dire Dawa Administration, eastern Ethiopia. Journal of Agricultural Extension and Rural Development, 8, 269-283.

Lasco, R. D., Espaldon, M. L. O., & Habito, C. M. D. (2016). Smallholder farmers’ perceptions of climate change and the roles of trees and agroforestry in climate risk adaptation: evidence from Bohol, Philippines. Agroforestry Systems, 90, 521-540.

Leitao, P., Henriques, S., Perez-Ibarra, I., Trujillo, M., García-Charton, J. A., & Vasconcelos, R. P. (2020). Shifting baselines in a Mediterranean small-scale fishery. Ocean & Coastal Management, 183, 104985.

Lovell, S., Johnson, A. E., Ramdeen, R., & McClenachan, L. (2020). Shifted baselines and the policy placebo effect in conservation. Oryx, 54, 383-391.

Lozano-Montes, H. M., Pitcher, T. J., & Haggan, N. (2008). Shifting environmental and cognitive baselines in the upper Gulf of California. Frontiers in Ecology and the Environment, 6, 75-80.

Lyver, P. O. B., Timoti, P., Richardson, S. J., & Gormley, A. M. (2021). Alignment of ordinal and quantitative species abundance and size indices for the detection of shifting baseline syndrome. Ecological Applications, 31, e02301.

Magadán-Díaz, M., & Rivas-García, J. I. (2022). Residents’ perception of sustainable tourism in protected mountain areas: the case of Asturias. Journal of Mountain Science, 19, 3597-3614.

Manandhar, S., Pratoomchai, W., Ono, K., Kazama, S., & Komori, D. (2015). Local people’s perceptions of climate change and related hazards in mountainous areas of northern Thailand. International Journal of Disaster Risk Reduction, 11, 47-59.

Mata-Lara, M., Garza-Pérez, J. R., Aranda-Fragoso, A., & de Almeida, P. S. A. (2018). Social alienation and environmental decline in a coral reef: challenges to coastal management in the Mexican Caribbean. Ocean & coastal management, 155, 30-39.

McClenachan, L., & Neal, B. (2023). Forgotten whales, fading codfish: Perceptions of ‘natural’ ecosystems inform visions of future recovery. *People and Nature*, 5, 699-712.

Mishra, A. K., & Pede, V. O. (2017). Perception of climate change and adaptation strategies in Vietnam: are there intra-household gender differences? International Journal of Climate Change Strategies and Management, 9, 501-516.

Muldrow, M., Parsons, E., & Jonas, R. (2020). Shifting baseline syndrome among coral reef scientists. Humanities and Social Sciences Communications, 7, 1-8.

Mustafa, G., Alotaibi, B. A., & Nayak, R. K. (2023). Linking climate change awareness, climate change perceptions and subsequent adaptation options among farmers. Agronomy, 13, 758.

Mwalusepo, S., Massawe, E. S., Affognon, H., Okuku, G. O., Kingori, S., Mburu, P. D., ... & Le Ru, B. P. (2015). Smallholder farmers’ perspectives on climatic variability and adaptation strategies in East Africa: the case of mount Kilimanjaro in Tanzania, Taita and Machakos Hills in Kenya. Journal of Earth Science & Climatic Change, 6, 1000313.

Ndambiri, H. K., Ritho, C. N., & Mbogoh, S. G. (2013). An evaluation of farmers' perceptions of and adaptation to the effects of climate change in Kenya. International Journal of Food and Agricultural Economics 1, 75-96.

Nyang’au, J. O., Mohamed, J. H., Mango, N., Makate, C., & Wangeci, A. N. (2021). Smallholder farmers’ perception of climate change and adoption of climate smart agriculture practices in Masaba South Sub-county, Kisii, Kenya. Heliyon, 7, e06789.

Ochieng, J., Kirimi, L., & Makau, J. (2017, November). Adapting to climate variability and change in rural Kenya: farmer perceptions, strategies and climate trends. Natural Resources forum, 41, 195-208.

Papworth, S. K., Rist, J., Coad, L., & Milner‐Gulland, E. J. (2009). Evidence for shifting baseline syndrome in conservation. Conservation letters, 2, 93-100.

Pita, P., Antelo, M., Hyder, K., Vingada, J., & Villasante, S. (2020). The use of recreational fishers’ ecological knowledge to assess the conservation status of marine ecosystems. Frontiers in Marine Science, 7, 242.

Pouso, S., Uyarra, M. C., & Borja, Á. (2018). Recreational fishers’ perceptions and behaviour towards cultural ecosystem services in response to the Nerbioi estuary ecosystem restoration. Estuarine, Coastal and Shelf Science, 208, 96-106.

Raghuvanshi, R., & Ansari, M. A. (2017). A study of farmers’ awareness about climate change and adaptation practices in India. International Journal of Applied Agricultural Sciences, 3, 154-160

Raza, M. A., & Ullah, S. (2022). Evaluating determinants of climate change perception among farming communities in district Mardan, Khyber Pakhtunkhwa-Pakistan. Journal of Agricultural Research, 60, 267-276.

Roco, L., Engler, A., Bravo-Ureta, B. E., & Jara-Rojas, R. (2015). Farmers’ perception of climate change in mediterranean Chile. Regional Environmental Change, 15, 867-879.

Sanogo, K., Binam, J., Bayala, J., Villamor, G. B., Kalinganire, A., & Dodiomon, S. (2017). Farmers’ perceptions of climate change impacts on ecosystem services delivery of parklands in southern Mali. Agroforestry systems, 91, 345-361.

Shitu, A. G., Olasehinde, T. S., & Kolawole, A. E. (2018). Driver factors of climate change household perception in southwest, Nigeria: an application of interaction probit model. Asian Journal of Multidisciplinary Studies, 6, 48-56.

Shrestha, U. B., Shrestha, A. M., Aryal, S., Shrestha, S., Gautam, M. S., & Ojha, H. (2019). Climate change in Nepal: a comprehensive analysis of instrumental data and people’s perceptions. Climatic Change, 154, 315-334.

Song, C. X., Liu, R. F., & Oxley, L. (2019). Do farmers care about climate change? Evidence from five major grain producing areas of China. Journal of Integrative Agriculture, 18, 1402-1414.

Li, C., Tang, Y., Luo, H., Di, B., & Zhang, L. (2013). Local farmers’ perceptions of climate change and local adaptive strategies: a case study from the Middle Yarlung Zangbo River Valley, Tibet, China. Environmental Management, 52, 894-906.

Tesfahunegn, G. B., Mekonen, K., & Tekle, A. (2016). Farmers’ perception on causes, indicators and determinants of climate change in northern Ethiopia: Implication for developing adaptation strategies. Applied Geography, 73, 1-12.

Teshome, H., Tesfaye, K., Dechassa, N., Tana, T., & Huber, M. (2021). Smallholder farmers’ perceptions of climate change and adaptation practices for maize production in Eastern Ethiopia. Sustainability, 13, 9622.

Teye, J. K., Yaro, J. A., & Bawakyillenuo, S. (2015). Local farmers’ experiences and perceptions of climate change in the Northern Savannah zone of Ghana. International Journal of Climate Change Strategies and Management, 7, 327-347.

Thi Lan Huong, N., Shun Bo, Y., & Fahad, S. (2017). Farmers’ perception, awareness and adaptation to climate change: evidence from northwest Vietnam. International Journal of Climate Change Strategies and Management, 9, 555-576.

Tofu, D. A. (2018). Smallholder farmers’ perception and responses to climate change and variability in West Shewa, Oromia, Ethiopia. Journal of Geography, Environment and Earth Science International, 18, 1-16.

Tuntipisitkul, P., Tsusaka, T. W., Kim, S. M., Shrestha, R. P., & Sasaki, N. (2021). Residents’ perception of changing local conditions in the context of tourism development: The case of Phuket Island. Sustainability, 13, 8699.

Turvey, S. T., Barrett, L. A., Yujiang, H. A. O., Lei, Z., Xinqiao, Z., Xianyan, W., ... & Ding, W. (2010). Rapidly shifting baselines in Yangtze fishing communities and local memory of extinct species. Conservation Biology, 24, 778-787.

van den Heuvel, L., & Rönnbäck, P. (2023). What you see isn’t always what you get: On how anglers’ fish stock perceptions are influenced by motivations, satisfaction and engagement. *Fisheries Research*, 258, 106519.

Veneroni, B., & Fernandes, P. G. (2021). Fishers’ knowledge detects ecological decay in the Mediterranean Sea. Ambio, 50, 1159-1171.

Venkatachalam, A. J., Price, A. R. G., Chandrasekara, S., Senaratna Sellamuttu, S., & Kaler, J. (2010). Changes in frigate tuna populations on the south coast of Sri Lanka: evidence of the shifting baseline syndrome from analysis of fisher observations. Aquatic Conservation: Marine and Freshwater Ecosystems, 20, 167-176.

Zapelini, C., da Silva, P. S., & Schiavetti, A. (2020). Shifting baseline syndrome highlighted by anecdotal accounts from snapper (*Ocyurus chrysurus*) fishery. Ethnobiology and Conservation, 9, 1-12.
