# Supplementary material for: Global synthesis indicates widespread occurrence of shifting baseline syndrome
Source: Bioscience. 2024 Aug 23;74(10):686–94. doi: 10.1093/biosci/biae068 (PMC11494512; doi:10.1093/biosci/biae068)
Supplement: biae068_Supplemental_Files [file biae068_supplemental_files.zip › Appendix 3.docx]

**Supplementary Appendix S3. Quality assessment using the Newcastle-Ottawa Scale (adapted for cross-sectional studies)**

| **Study** | **Representativeness of the sample** | **Sample size** | **Non-respondents** | **Comparability** | **Total score** |
| --- | --- | --- | --- | --- | --- |
| Agwu et al. (2018) | 1 | 1 | 0 | 2 | 4 |
| Ainsworth et al. (2008) | 0 | 0 | 0 | 0 | 0 |
| Akano et al. (2023) | 1 | 0 | 0 | 2 | 3 |
| Alessa et al. (2008) | 1 | 0 | 0 | 0 | 1 |
| Alfonso et al. (2017) | 1 | 0 | 0 | 2 | 3 |
| Ali et al. (2017) | 1 | 0 | 0 | 2 | 3 |
| Almojil (2021) | 1 | 0 | 0 | 0 | 1 |
| Amadou et al. (2015) | 1 | 0 | 0 | 2 | 3 |
| Amoutchi et al. (2021) | 1 | 1 | 0 | 2 | 4 |
| Ansari et al. (2018) | 0 | 0 | 0 | 2 | 2 |
| Apata (2011) | 1 | 0 | 0 | 2 | 3 |
| Assaye (2016) | 1 | 0 | 0 | 2 | 3 |
| Ayal and Leal Filho (2017) | 1 | 0 | 0 | 2 | 3 |
| Bao and Drew (2016) | 0 | 0 | 0 | 0 | 0 |
| Barbosa-Filho et al. (2020) | 1 | 1 | 0 | 0 | 2 |
| Bender et al. (2013) | 1 | 0 | 0 | 0 | 1 |
| Bender et al. (2014) | 1 | 0 | 0 | 0 | 1 |
| Bobadoye et al. (2020) | 1 | 0 | 0 | 2 | 3 |
| Braga et al. (2022) | 1 | 0 | 0 | 0 | 1 |
| Bunce et al. (2008) | 1 | 0 | 0 | 0 | 1 |
| Coster and Adeoti (2021) | 1 | 1 | 0 | 2 | 4 |
| Debela et al. (2015) | 1 | 1 | 0 | 2 | 4 |
| Deressa et al. (2011) | 1 | 0 | 0 | 2 | 3 |
| Fatuase et al. (2014) | 1 | 0 | 0 | 2 | 3 |
| Fernández-Llamazares et al. (2015) | 1 | 0 | 0 | 0 | 1 |
| Frezza and Clem (2015) | 0 | 0 | 0 | 0 | 0 |
| Funatsu et al. (2019) | 0 | 0 | 0 | 0 | 0 |
| Giglio et al. (2015) | 0 | 0 | 0 | 0 | 0 |
| Guodaar et al. (2017) | 0 | 1 | 0 | 2 | 3 |
| Habtemariam et al. (2016) | 1 | 0 | 0 | 2 | 2 |
| Jabik et al. (2022) | 1 | 1 | 0 | 2 | 4 |
| Jones et al. (2020) | 0 | 0 | 0 | 2 | 2 |
| Katikiro (2014) | 1 | 0 | 0 | 0 | 1 |
| Kawadia and Tiwari (2017) | 1 | 0 | 0 | 0 | 1 |
| Kidanu et al. (2016) | 1 | 0 | 0 | 0 | 1 |
| Lasco et al. (2016) | 1 | 0 | 0 | 2 | 3 |
| Leitao et al. (2020) | 1 | 0 | 0 | 0 | 1 |
| Li et al. (2013) | 1 | 0 | 0 | 0 | 1 |
| Lovell et al. (2020) | 1 | 0 | 0 | 0 | 1 |
| Lozano-Montes et al. (2008) | 1 | 0 | 0 | 0 | 1 |
| Lyver et al. (2021) | 0 | 0 | 0 | 0 | 0 |
| Magadán-Díaz and Rivas-García (2022) | 1 | 0 | 0 | 0 | 1 |
| Manandhar et al. (2015) | 1 | 1 | 0 | 2 | 4 |
| Mata-Lara et al. (2018) | 1 | 1 | 0 | 0 | 2 |
| McClenachan and Neal (2023) | 0 | 0 | 0 | 0 | 0 |
| Mishra and Pede (2017) | 1 | 0 | 0 | 2 | 3 |
| Muldrow et al. (2020) | 1 | 0 | 0 | 0 | 1 |
| Mustafa et al. (2018) | 1 | 0 | 0 | 2 | 3 |
| Mwalusepo et al. (2015) | 1 | 0 | 0 | 2 | 3 |
| Ndambiri et al. (2013) | 1 | 0 | 0 | 2 | 3 |
| Nyang'au et al. (2021) | 1 | 0 | 0 | 2 | 3 |
| Ochieng et al. (2017) | 1 | 0 | 0 | 2 | 3 |
| Papworth et al. (2009) | 0 | 0 | 0 | 2 | 2 |
| Pita et al. (2020) | 1 | 0 | 0 | 0 | 1 |
| Pouso et al. (2018) | 0 | 0 | 0 | 0 | 0 |
| Raghuvanshi et al. (2017) | 1 | 0 | 0 | 0 | 1 |
| Raza et al. (2022) | 1 | 0 | 0 | 2 | 3 |
| Roco et al. (2015) | 1 | 0 | 0 | 2 | 3 |
| Sanogo et al. (2017) | 1 | 1 | 0 | 0 | 2 |
| Shitu et al. (2018) | 1 | 0 | 0 | 2 | 3 |
| Shrestha et al. (2019) | 1 | 0 | 0 | 2 | 3 |
| Song et al. (2019) | 1 | 0 | 0 | 2 | 3 |
| Tesfahunegn et al. (2016) | 1 | 1 | 0 | 2 | 4 |
| Teshome et al. (2021) | 1 | 0 | 0 | 2 | 3 |
| Teye and Yaro (2015) | 1 | 0 | 0 | 2 | 3 |
| Thi Lan Huong et al. (2017) | 1 | 0 | 0 | 2 | 3 |
| Tofu (2018) | 1 | 0 | 0 | 2 | 3 |
| Tuntipisitkul et al. (2021) | 0 | 1 | 0 | 0 | 1 |
| Turvey et al. (2010) | 0 | 0 | 0 | 2 | 2 |
| Veneroni and Fernandes (2021) | 0 | 0 | 0 | 0 | 0 |
| Venkatachalam et al. (2010) | 1 | 0 | 0 | 0 | 1 |
| Zapelini et al. (2020) | 1 | 0 | 0 | 0 | 1 |
| van den Heuvel and Rönnbäck (2023) | 1 | 0 | 0 | 0 | 1 |
